# Emerging mechanistic trends and clinical efficacy for methotrexate: Applications to inflammatory bowel disease

**DOI:** 10.1016/j.pharmr.2026.100132

**Published:** 2026-03-19

**Authors:** Jeehyun Karen You, Theresa T. Pizarro, Tommaso L. Parigi

**Affiliations:** 1 Departments of Pathology and Biochemistry, Case Western Reserve University School of Medicine, Cleveland, Ohio; 2 Department of Pathology, Case Western Reserve University School of Medicine, Cleveland, Ohio; 3 Università Vita-Salute and Department of Gastroenterology, IRCCS Ospedale San Raffaele, Milan, Italy

## Abstract

Methotrexate (MTX) was among the first steroid-sparing agents introduced for the treatment of inflammatory bowel disease (IBD). Its efficacy is well established for Crohn’s disease, though studies of its use in ulcerative colitis have largely reported negative results, with only some, although inconsistent, findings suggesting limited benefit. In this review, we provide a comprehensive and up-to-date evaluation of MTX’s use in IBD, including its key mechanisms of action(s), therapeutic value, as well as emerging targets that underscore the complexity of this drug and the biological landscape it alters. Despite its longstanding use, the full spectrum of MTX’s effects contributing to its efficacy in IBD remains incompletely understood. Although multiple pathways have been implicated, the relative importance of each remains nebulous, and additional, unidentified functions may play a role, particularly in contexts not pertaining to immunomodulation. We highlight recent findings that poise MTX as an unexpected but promising agent for mucosal healing. We also provide a detailed evaluation of clinical studies, encompassing randomized controlled trials and observational data, highlighting MTX’s effectiveness, differences in route of administration, safety profile, and limitations as they pertain to the management of IBD. As therapeutic targets for IBD evolve, we discuss MTX’s future positioning by exploring clinical perspectives regarding its utility and examine the latest evidence that indicates there may be novel, previously unexplored, therapeutic potential. By bridging mechanistic insights with clinical evidence, this review underscores MTX’s enduring, albeit niche, position in IBD therapy and highlights key areas for future investigation to optimize its use.

**Significance Statement:**

Methotrexate remains a valuable, though underutilized, therapeutic option for the management of inflammatory bowel disease. This review provides a comprehensive overview of methotrexate’s pharmacology, integrating emerging mechanistic insights and clinical data to reframe its role beyond immunomodulation, particularly at the gastrointestinal mucosal interface, thereby identifying novel avenues for future research that may expand its clinical utility in inflammatory bowel disease.

## Graphical Abstract

MTX pharmacology during IBD. Current data regarding pharmacology of MTX is primarily derived from patients with RA and models of inflammatory arthritis. As such, relative contributions of well established anti-inflammatory mechanisms targeting immune cells, specifically in IBD, are unclear. However, emerging evidence indicates that MTX-mediated alterations to gut microbiome and intestinal epithelial barrier may play critical roles in IBD. Cumulative evidence indicates pharmacology of MTX is likely unique and distinct from that canonically associated with RA. Abbreviation: DAMPA, 2,4-diamino-N^10^-methylpteroic acid.

**Figure:**
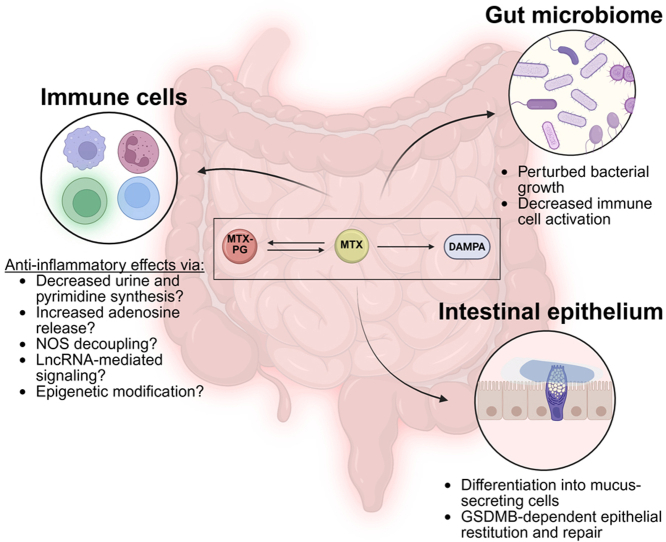

## I Introduction

Inflammatory bowel disease (IBD) comprises 2 major disorders, ulcerative colitis (UC) and Crohn’s disease (CD), both of which are chronic, relapsing diseases of the gastrointestinal (GI) tract. These 2 clinical subtypes differ in location and extent of disease, whereby CD can affect the entire length of the GI tract, but predominantly involves the terminal ileum and/or colon; CD inflammation manifests as patchy, segmental, and transmural lesions. On the other hand, UC is limited to the colon and primarily affects the superficial layers of the gut wall, with the presence of mucosal ulcerations. Despite recent advances in therapeutic options for IBD, the overall response to treatment remains relatively disappointing, highlighting the need for new drugs and/or more effective combinations of existing ones. In recent years, much research has been devoted to novel target identification and drug discovery. However, older drugs, such as thiopurines, glucocorticoids, and methotrexate (MTX), remain fundamental for the treatment of IBD.

MTX was originally introduced as a chemotherapeutic agent in the 1940s, but its use was at high doses.^1^ It has since been repurposed and is now primarily used at lower doses for the treatment of autoimmune and inflammatory diseases, including CD.^2^^,^^3^ In the context of IBD, the therapeutic properties of MTX are largely attributed to its immunomodulatory and anti-inflammatory effects via folate antagonism, adenosine release, and reduced production of proinflammatory cytokines. However, the relative contribution of each of these mechanisms, and the extent of biological processes modulated by MTX, are incomplete because most studies focus on its effects on immune cells. Moreover, existing data predominantly addresses rheumatoid arthritis (RA) pathophysiology, not IBD. However, emerging evidence indicates that MTX may alter the gut microbiome and promote mucosal healing at the intestinal epithelial barrier, with important considerations for enterocyte biology. Such advancements in our understanding of MTX pharmacology highlight a need to critically appraise the role of MTX in the management of IBD. In this review, we contextualize MTX’s pharmacology in IBD pathophysiology and summarize the clinical data on the efficacy and safety in patients with IBD. We also explore the implications of MTX in the GI tract and discuss future perspectives, including potential ways to optimize MTX therapy for IBD. In short, we aim to provide new insights into MTX pharmacology, which has long been part of the therapeutic armamentarium used to treat patients living with IBD.

## II Pharmacology of methotrexate

**Fig. 1:**
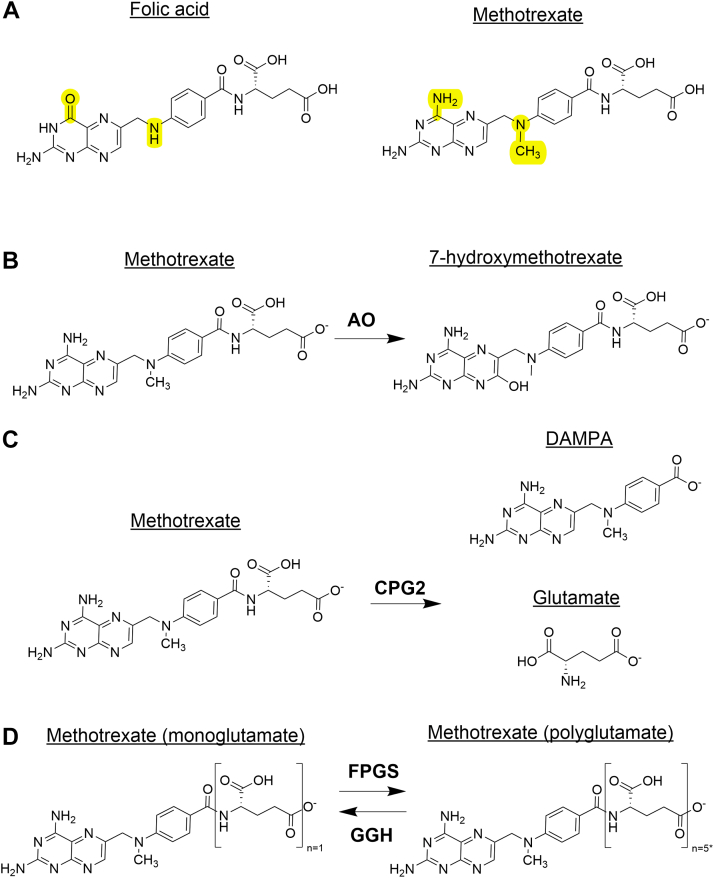
Chemical structures of folic acid, MTX, and key MTX-derived molecules. (A) MTX, highly comparable, structurally, to folic acid (differing moieties highlighted in yellow) is (B) partially oxidized by hepatic aldehyde oxidase (AO), and (C) deglutamated to 2,4-diamino-N^10^-metnylpteroate (DAMPA) by the intestinal microflora, whereas carboxypeptidase G2 (CPG2), also known as glucarpidase (a recombinant bacterial enzyme), mediates MTX deglutamation into DAMPA and glutamate. (D) Within cells, MTX (a monoglutamate) undergoes serial polyglutamylation to form active MTX polyglutamates. During MTX polyglutamylation, additional glutamate residues are added to terminal glutamate moieties by FPGS; GGH mediates the opposite reaction (deglutamation). ∗Addition of up to 7 glutamate resides has been reported, but cumulatively, native MTX (monoglutamate) up to MTX pentaglutamate (*n* = 5) account for 99.6% of total intracellular MTX-PG.^4^ Structures generated by Revvity Signals Software, Inc; ChemDraw Professional, Version 25.0.2; PerkinElmer Informatics, 2025.

MTX is a weak bicarboxylic acid and an analog of folic acid (Fig. 1A)^4^ with dose-dependent medical applications. High-dose MTX (≥500 mg/m^2^) was first introduced in the 1940s as a cytotoxic agent; its use as a chemotherapeutic agent has since diminished, although it is currently used to treat a specific subset of adult and pediatric cancers, including acute lymphoblastic leukemia, osteosarcoma, and central nervous system lymphomas.^5^ Low-dose MTX (up to 25–30 mg/wk), on the other hand, is commonly used to treat various forms of inflammatory arthritis, as well as other autoimmune and inflammatory diseases. In fact, low-dose MTX is currently the gold standard for treating RA.^6^, ^7^, ^8^ In this section, we will: (1) explore key aspects of low-dose MTX pharmacokinetics (PK)s, (2) discuss the diverse mechanisms of action and subsequent biological effects mediated by MTX, and (3) summarize key mechanisms of MTX resistance. It is important to note, however, that much of the current evidence regarding the PK and pharmacodynamic properties of MTX are limited to its use in RA and less is known about MTX’s pharmacology specifically in patients with IBD.

### A Clinical pharmacokinetics

Clinical PKs of low-dose MTX have long been established, with key studies published over 30 years ago. MTX absorption was first described to occur in the small intestine.^9^ Subsequent studies using organ-cultured endoscopic biopsy specimens of intestinal mucosa (proximal small intestinal, specifically) from normal subjects demonstrated 2 distinct fluxes of MTX: a folic acid–sensitive flux whose contribution is about one-third of the total flux, and a folic acid-insensitive flux that accounts for the rest. These results demonstrate that folic acid-insensitive flux is a major component of the total flux of MTX in human proximal small intestines, indicating a heterogeneity of MTX transport.^10^ Given the significant interindividual variability reported for oral MTX absorption, the dual-flux system may be an important contributing factor explaining these differences. In terms of disease-specific low-dose MTX PK parameters, data from key studies using patients with RA and IBD are shown in Table 1.^11^, ^12^, ^13^ Although most studies have been conducted in RA, 2 studies in patients with IBD, albeit analyzing limited cohorts^12^ (*n* ≤ 10), suggest interesting trends specific to IBD. Moshkowitz et al^12^ report that the mean maximum plasma concentration (C_max_) values for patients with CD versus UC versus RA are similar after oral administration of MTX (12.5 mg); however, the mean time to reach C_max_ (t_max_), varies between groups. Specifically, patients with CD show a significantly lower t_max_ when compared with patients with UC. Moreover, a negative correlation exists between MTX dosage and C_max_ in patients with CD, whereby C_max_ decreases with increased MTX dosage/kg. These results, although limited by the aforementioned small sample size, suggest that patients with CD may have a distinct PK profile for MTX. Egan et al^13^ examined intestinal PK in patients with IBD, in addition to the standard systemic PK, after administration of 15 or 25 mg s.c. MTX. The study specifically examines MTX in the luminal extracellular fluid and mucosal MTX of biopsied rectal tissues. Although luminal rectal and mucosal MTX concentrations were not significantly different between subjects receiving 15 or 25 mg/wk, mucosal MTX was significantly higher than luminal MTX. Importantly, MTX concentrations were found to be within the pharmacologically active range. The relatively high concentration of MTX found in the rectal mucosa suggests that the drug may not only act locally at the site of disease, but likely reaches rectal tissue from the circulation. However, the study did not find a concentration-response relationship in terms of rectal MTX concentration (both luminal and mucosal) and severity of inflammation.^13^ According to the authors, this may be attributable to mucosal hyperemia caused by inflammation. Given the high concentrations of MTX in the rectal mucosa, Caco-2 intestinal epithelial cells (IECs) were treated with the MTX concentrations (both trough and peak) found within the rectal mucosa. These concentrations were found to be active against Caco-2 IEC growth.^13^ These findings, however, are not generalizable to patients with IBD as there are significant limitations to this study. First and foremost, MTX concentration(s) were not evaluated after oral administration (a well established route of administration in patients with IBD) or at the proximal small intestines, the predominant site of MTX absorption. Second, the small sample size deters any conclusive PK characterizations specific to patients with IBD. Given that patients with IBD likely have a PK profile distinct from normal healthy subjects, additional studies are warranted to better characterize the PK parameters for IBD.

**Table 1:** Low-dose MTX clinical PKs Values are expressed as aggregate means.

Clinical Indication	Dose (mg)	Route of Administration	C_max_ (*μ*mol/L)	t_max_ (h)	V_ss_ (L/kg)	V_z_ (L/kg)	t_1/2*β*_ (h)	CL (L/h)	MRT (h)
RA∗	16.3	p.o.	0.72	1.16	0.59	—	5.95	8.7	—
RA∗	12.5	i.v.	—	—	—	0.85	6.37	6.7	4.7
RA∗	10	i.m.	0.62	0.89	1.02	1.43	8.25	7.06	8.0
IBD^†^	15	s.c.	1.14	0.85	—	—	2.8, 64^‡^	5.8	—
CD^†^	15	s.c.	1.17	0.88	—	—	3.2, 55^‡^	5.2	—
UC^†^	15	s.c.	1.12	0.83	—	—	2.5, 70.3^‡^	6.1	—
IBD^†^	12.5	p.o.	0.63	1.62	—	—	—	—	—
CD^†^	12.5	p.o.	0.59	1.4	—	—	—	—	—
UC^†^	12.5	p.o.	0.69	1.9	—	—	—	—	—

CL, total body clearance; i.m., intramuscular; i.v., intravenous; MRT, mean residence time in the body; p.o., by mouth; s.c., subcutaneous; t_1/2*β*_, terminal elimination half-life; t_max_, time to C_max_; V_ss_, volume of distribution at steady-state; V_z_, volume of distribution during terminal elimination phase.

^∗^Summary data aggregated from patients with RA.^11^

^†^Summary data aggregated from patients with IBD,^12^^,^^13^ further subdivided into CD and UC.

^‡^First and second elimination half-lives due to biphasic elimination kinetics.

The well established absorption, distribution, metabolism, and excretion characteristics of MTX demonstrate not only the complexity of MTX pharmacology, but highlight specific considerations for patients with IBD. Key absorption, distribution, metabolism, and excretion characteristics are summarized in Table 2.^11^^,^^14^ MTX is converted by hepatic aldehyde oxidase to 7-hydroxy-methotrexate (7-OH-MTX) (Fig. 1B), which is poorly water soluble and weakly active compared with the parent drug.^11^ Although less than 10% of MTX is hydroxylated, plasma concentrations of 7-OH-MTX usually exceed those of MTX within a few hours of drug administration. Thus, 7-OH-MTX is the major circulating metabolite of MTX. However, studies evaluating the pharmacodynamic properties of 7-OH-MTX have been limited to reporting its immunomodulatory and antiproliferative functions. Of note, MTX and 7-OH-MTX may compete for entry into cells and subsequent transformation to polyglutamate derivatives, which are the active forms of MTX responsible for its immunomodulatory and antiproliferative effects.^11^

**Table 2:** Key absorption, distribution, metabolism, and excretion characteristics of low-dose MTX As summarized by Shen and Azarnoff^14^ and Bannwarth et al.^11^

Absorption	Distribution	Metabolism	Excretion
• Follows either zero or first-order kinetics • Mainly absorbed in proximal jejunum • Displays 2 distinct fluxes across proximal small intestinal mucosa: 1/3—folic acid–sensitive flux2/3—folic acid-insensitive flux • Considerable interindividual variability • Low intraindividual variability over long periods • Up to 1/3 of administered oral dose may be metabolized by gut flora before drug absorption • i.m./i.v./s.c. vs p.o. administration reported to result in faster absorption rates and higher serum concentrations	• Distributes to extravascular compartments • Transported into cells by RFC1, whereas free nonglutamated MTX removed from cells by ABC transporters • Circulating MTX binds almost exclusively to albumin fraction of plasma proteins (42%–57%) • Bound fraction of MTX exhibits large interindividual variability • Percentages of drug bound to serum of healthy vs patients with RA are comparable	• First-pass metabolism is negligible • Nearly 1/3 MTX may be metabolized by CPG2 from intestinal bacteria and results in formation of DAMPA • Converted by hepatic AO to 7-OH-MTX • 7-OH-MTX is major circulating metabolite with extensive protein binding (serum albumin; 91%–93%) • Can be reversibly transformed into polyglutamate derivative (MTX-PG) by FPGS and GGH • MTX-PG concentrations in RBCs are stable, whereas serum concentrations rapidly fall below limit of detection • IECs have very active form of hydrolase enzyme; consequently do not accumulate MTX-PG	• Mainly excreted by kidney as intact drug, regardless of route of administration (60%–90% during 24 h urinary recovery) • Glomerular filtration is dominant pathway • Drug additionally undergoes bidirectional transport within renal tubules, with active secretory process using general organic acid transporters • Renal clearance decreased by concomitant administration of organic acids, such as salicylate • Variable amounts (10%–30%) eliminated by active biliary secretion; thus, available for enterohepatic recirculation • Correlates with endogenous creatinine clearance, demonstrating importance of renal function

AO, aldehyde oxidase; CPG2, carboxypeptidase G2; DAMPA, 2,4-diamino-N^10^-metnylpteroate; i.m., intramuscular; i.v., intravenous; p.o., by mouth; RFC1, reduced folate carrier *α*; s.c., subcutaneous.

Upon oral administration, MTX can be deglutamated by the intestinal microflora to 2,4-diamino-*N*^10^–methylpteroate and glutamate (Fig. 1C),^15^ 2 noncytotoxic metabolites that are eliminated primarily by the liver.^16^ In fact, MTX metabolism can be ameliorated in germ-free mice and by pretreatment with antibiotics.^17^ The occurrence of in vivo bacterial metabolism is further supported by work showing that pretreatment of patients with kanamycin leads to a substantial increase in plasma levels and recovery of MTX after oral administration.^14^ Interestingly, pretreatment with neomycin, which is poorly absorbed when administered orally, is associated with reduced plasma MTX concentrations. These results may be attributed to the established ability of neomycin to cause malabsorption of many drugs and nutrients; moreover, these studies, performed by Shen and Azarnoff,^14^ used different routes of administration: intravenous for kanamycin and by mouth for neomycin. Given that aminoglycosides are absorbed well when delivered intramuscularly or intravenously and less so orally, the biological effects are likely different across the 2 studies.

Carboxypeptidase G2, also known as glucarpidase, is a bacterial enzyme that rapidly lowers MTX concentrations via deglutamation (Fig. 1C). Glucarpidase rapidly metabolizes circulating MTX to reduce plasma MTX concentrations by >95%, within 15 minutes of administration.^18^ Given that MTX is primarily excreted in its unchanged form in urine^11^^,^^14^ (Table 2), glucarpidase is clinically indicated for patients with delayed MTX clearance due to impaired renal function and for patients experiencing high-dose MTX-induced acute kidney injury.^18^^,^^19^

**Fig. 2:**
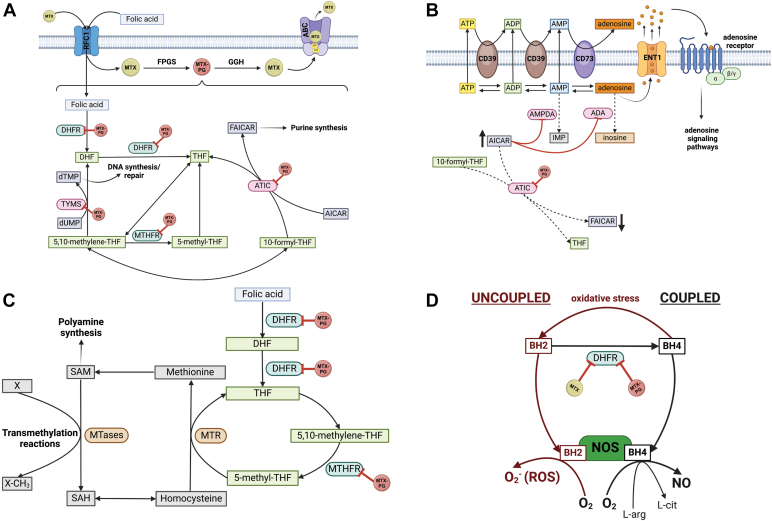
Key biochemical pathways targeted by MTX. (A) MTX enters cells via reduced folate carrier *α* (RFC1), before undergoing polyglutamylation. MTX-PG is trapped within cells and can undergo glutamylation/deglutamylation by FPGS/GGH; only MTX can be exported from cells via ABC transporters. MTX-PG (rust-colored circles) can inhibit multiple folate-dependent enzymatic reactions, including those mediated by DHFR, MTHFR, TYMS, and ATIC, resulting in decreased de novo purine synthesis and DNA synthesis/repair. (B) ATIC inhibition by MTX-PG results in elevated intracellular AICAR levels that inhibit AMP deaminase (AMPDA) and ADA, resulting in elevated levels of extracellular adenosine (through CD39- and CD73-mediated extracellular adenosine production and intracellular adenosine accumulation/release) via equilibrative nucleoside transporter 1 (ENT1) and increased adenosine receptor signal transduction. (C) MTX-PG inhibits key components of the 1-carbon cycle; decreased levels of 5-methyl-THF results in decreased methionine synthase (MTR) activity. The consequent decrease in methionine and methyl group donor S-adenosylmethionine (SAM) levels diminish intracellular transmethylation reactions and polyamine synthesis. (D) MTX and MTX-PG inhibit DHFR-mediated BH4 production, which critically mediates coupling between oxygen reduction and L-Arg oxidation for NO production by NOS. With diminished BH4 and increased BH2 levels, electron transfer and reduction of oxygen are uncoupled from L-Arg oxidation, resulting in generation of superoxide anion (O_2_^−^) and other reactive oxygen species (ROS). ADA, adenosine deaminase; DHF, dihydrofolate; dUMP, deoxyuridine monophosphate; FAICAR, 5-formamidoimidazole-4-carboxamide ribotide; L-cit, L-citrulline; MTase, methyltransferases; SAH, S-adenosylhomocysteine. Created in BioRender. Pizarro, T. (2025) https://BioRender.com/smmixvl.

MTX is transported into cells by reduced folate carrier *α* (RFC1), a protein encoded by the gene, *SLC19A1*,^20^^,^^21^ whereas free nonglutamated MTX is removed from cells by transporters belonging to the ATP-binding cassette (ABC) family (Fig. 2A).^22^ MTX, such as folic acid, contains a single glutamate moiety. Upon its entry into cells, up to 4 additional glutamates are added by folylpolyglutamyl synthase (FPGS), resulting in polyglutamylated MTX with up to 5 glutamates (MTX-PG) (Fig. 1D). The most prevalent type of polyglutamate species is MTX-PG_3_ (3 glutamates).^23^ This is a reversible reaction, as *γ*-glutamyl hydrolase (GGH) sequentially removes terminal glutamate moieties to return MTX-PG to MTX (Fig. 1B). Polyglutamylation is a critical step of MTX metabolism, as it not only potentiates its pharmacological actions, but traps MTX inside cells.^24^ MTX polyglutamylation primarily occurs in red blood cells (RBCs),^25^ indicating that RBCs significantly contribute to the bioavailability and distribution of MTX during long-term therapy. RBCs, however, are not the only cells that can generate MTX-PGs. Indeed, MTX-PGs are also found in neutrophils, peripheral blood mononuclear cells, hepatocytes, and synoviocytes, after oral administration.^26^ MTX metabolism, however, is likely cell type–specific; for example, peripheral blood mononuclear cells primarily generate short-chain MTX-PG (up to 2 glutamates added), which is associated with the unfavorable kinetics and low activity of FPGS.^27^ Some data also suggest that route of administration affects MTX polyglutamylation. Specifically, the concentration of long-chain MTX-PG (3–5 glutamates added) is significantly higher than that of short-chain MTX-PG in patients with RA treated with subcutaneous MTX. In contrast, patients treated with oral MTX have higher concentrations of short-chain MTX-PG compared with long-chain MTX-PG.^4^ After commencing MTX, or changing dosage, MTX-PG concentrations reach steady-state levels in RBCs after 6 months of continued therapy, with considerable interpatient variability. Accordingly, the MTX dose for appropriate clinical management of RA can vary and may be difficult to predict. In IBD, higher doses of many drugs are often associated with improved therapeutic efficacy. Because MTX levels can fluctuate or take time to stabilize, several studies propose to measure MTX-PG concentrations as potential biomarkers to guide dose adjustments. This approach, known as therapeutic drug monitoring, aims to optimize efficacy by tailoring the dose to an individual’s PK profile. Although not currently used in clinical practice, the potential for MTX-PG-based therapeutic drug monitoring in IBD will be discussed in further detail in Clinical perspectives.

The progressive accumulation of polyglutamated forms and the delayed PK profile explain, at least in part, the slow onset of MTX’s clinical effects. In the setting of IBD, the full effect of MTX is typically seen around 12 to 14 weeks, making it a relatively slow-acting drug, often requiring concomitant administration of faster-acting agents, such as steroids, for prompt control of symptoms. For comparison, the efficacy of MTX in RA is typically observed a few weeks earlier.^25^^,^^28^ Additionally, it is important to note that IECs express a potent hydrolase, which results in effective deglutamation of intracellular MTX-PG (Table 2).^11^ As such, MTX-PG is unlikely to be the form of MTX found within IECs, independent of route of administration. Because the gut microbiome can deglutamylate MTX to 2,4-diamino-*N*^10^–methylpteroate, additional studies are needed to better define MTX metabolism specific to the intestinal mucosal barrier. Contributions from IECs and the gut microbiome may confer a MTX metabolite profile unique from those of RBCs, immune cells, hepatocytes, and synoviocytes.

### B Mechanisms of action

The mechanisms of action of MTX are incompletely understood, although it is generally accepted that MTX, once polyglutamylated, potently inhibits various folate-dependent enzymes. In fact, MTX-PG is 10- to 2500-times more potent as an inhibitor of key folate-dependent enzymes compared with MTX.^29^ Key cellular effects include (but are not likely limited to): inhibition of purine and pyrimidine synthesis, adenosine release, inhibition of transmethylation reactions and polyamine synthesis, and nitric oxide synthase (NOS) uncoupling. These well established pharmacodynamic properties, however, are largely based on clinical, in vivo, and in vitro data derived from RA or various models of inflammatory arthritis. The specific relevance of these canonical mechanisms of action to IBD remains understudied.

#### 1 Purine and pyrimidine synthesis

MTX, specifically MTX-PG, is a competitive inhibitor of dihydrofolate reductase (DHFR), thereby preventing the regeneration of tetrahydrofolate (THF) from dihydrofolate. THF is essential to generate folate cofactors required for de novo purine and pyrimidine synthesis to facilitate DNA and RNA synthesis/repair. THF, once converted to 5,10-methylene-THF, is an essential carbon donor and coenzyme for the methylation of deoxyuridine monophosphate to deoxythymidine monophosphate by thymidylate synthase (TYMS). TYMS is also directly inhibited by MTX-PG (Fig. 2A). Diminished deoxythymidine monophosphate levels result in impaired DNA synthesis/repair, a well established antineoplastic mechanism mediated by MTX.^30^ The inhibition of de novo purine synthesis, on the other hand, is primarily attributed to the inhibition of 5-aminoimidazole-4-carboxamide ribonucleotide (AICAR) transformylase (ATIC), which catalyzes the last committed step in de novo purine biosynthesis (Fig. 2A). Although a decrease in de novo purine and pyrimidine synthesis was initially thought to confer MTX’s anti-inflammatory effects, as lymphocytes in inflamed tissues are more dependent on de novo synthesis compared with other cells, cumulative evidence challenges this paradigm Indeed, MTX is effective in treating RA, even when concurrent folic acid or folinic acid supplementation prevents reductions in peripheral leukocyte counts, and the anti-inflammatory effects of MTX are at least partially independent from inhibition of lymphocyte cell proliferation.^8^ In fact, the decrease in inflammatory mediators, such as tumor necrosis factor (TNF), is greater than the reduction of immune cell infiltration. Mechanistically, MTX is reported to regulate nuclear factor-*κ*B (NF-*κ*B) activity in proinflammatory macrophages through a TYMS/p53 axis.^31^ As a pivotal mediator of inflammatory responses, NK-*κ*B has been implicated in the pathogenesis of various inflammatory diseases, including RA and IBD.^32^ NF-*κ*B regulation, therefore, may be a key downstream consequence of MTX’s effects on nucleotide synthesis. Overt inhibition of cellular proliferation, especially at higher doses typically used in oncology, more likely mediates the toxic effects of MTX, resulting in leukopenia, mucositis, and hair loss, which are almost exclusively seen with high-dose MTX use in patients with cancer, but not in patients with IBD on low-dose MTX.^8^^,^^24^

#### 2 Adenosine release

As described above, MTX-PG potently inhibits ATIC, thereby diminishing de novo purine synthesis.^29^^,^^33^ The consequent accumulation of AICAR inhibits AMP deaminase and adenosine deaminase, resulting in the release of adenine nucleotides (ie, AMP, ADP, and ATP) into the extracellular space. Once adenine nucleotides are released from the cell, they are hydrolyzed to adenosine by ectonucleoside triphosphate dephosphorylase (CD39; ATP to AMP) and ecto-5’-nucleotidase (CD73; AMP to adenosine) (Fig. 2B).^8^^,^^34^ Adenosine activates a family of G-protein-coupled receptors, namely adenosine receptors (A_1_, A_2A_, A_2B_, and A_3_), all of which have potent inhibitory effects across various inflammatory cell types (ie, neutrophils, macrophages, T cells, endothelial cells, and fibroblast-like synoviocytes) canonically identified in RA (reviewed by Cronstein and Sitkovsky^35^).

The adenosine system, however, plays a complex and nuanced role in IBD. Over the years, several preclinical studies highlighted the importance of adenosine in intestinal homeostasis. In addition to counteracting mucosal inflammation, selective adenosine receptor activation is reported to ameliorate other GI-related disorders, such as increased gut motility and diarrhea, as well as enteric visceral pain. However, a critical assessment of the clinical implications of pharmacologically targeting the adenosine system to manage IBD is complicated by the heterogeneity of the in vivo and in vitro models used to study adenosine signaling (discussed by Antonioli et al^36^). Moreover, the downstream effects of adenosine signaling in IBD may differ from RA. In fact, cumulative evidence indicates that the biological effect(s) of adenosine are receptor-specific in IBD. For one, the A_2A_ receptor can promote anti-inflammatory signaling, which is often impaired in IBD. In the colonic mucosa of patients with UC, A_2A_ receptor is posttranscriptionally downregulated by miR-16, resulting in decreased inhibition of NF-*κ*B-mediated proinflammatory signaling.^37^ On the other hand, the A_2B_ receptor, the predominant adenosine receptor expressed in the colon, is reported to induce proinflammatory signaling. In murine models of experimental colitis, A_2B_ receptor antagonism has not only been shown to suppress inflammatory infiltration in the colonic mucosa, but also improves intestinal epithelial architecture and barrier function.^38^ Additionally, A_3_ receptor is proposed to have both anti-inflammatory and proinflammatory properties in murine models of experimental colitis, as well as in studies using mucosal biopsies or peripheral blood mononuclear cells derived from patients with IBD.^38^ Given that the biological effect(s) of adenosine is likely disease-specific, the anti-inflammatory contributions of MTX-PG-mediated adenosine release in IBD requires further study. In fact, one study reports that either 15 or 25 mg s.c. MTX does not alter plasma or rectal adenosine concentrations. It is important to note, however, that the study only examined 10 patients with IBD, with all patients previously managed with weekly MTX administration (10–240 months) and concomitant medications (prednisone and 5-amino salicylic acid).^39^ It is, therefore, plausible that adenosine levels may have previously reached steady-state in patients examined during the study.

Interestingly, MTX also increases adenosine release by B cells, leading to diminished immunization against therapeutic monoclonal antibodies (ie, anti-TNF antibodies, such as infliximab [IFX], an immunosuppressive drug commonly used to treat IBD) in a B-cell activating factor (BAFF)-dependent manner.^40^ Although these studies were conducted using mouse models, they provide insight into the mechanistic basis for MTX-mediated antidrug antibody suppression in patients receiving combined MTX and anti-TNF therapy, including those with IBD. Based on these observations, several clinical studies in IBD (discussed in sections Maintenance of remission and Combination approaches with methotrexate) have been performed to investigate the benefits of MTX addition to other biologics, particularly IFX.

#### 3 Transmethylation reactions and polyamine synthesis

As described above, DHFR and methylenetetrahydrofolate reductase (MTHFR), are critical in generating key folate cofactors, namely THF and 5-methyl-THF. 5-methyl-THF is a coenzyme needed for recycling homocysteine to methionine via methyl tetrahydrofolate-homocysteine methyltransferase, also known as methionine synthase. Methionine, in turn, can be converted to S-adenosylmethionine, a key methyl donor for various transmethylation reactions, including DNA methylation (Fig. 2C).^34^ In patients with RA, polyamines, such as spermine and spermidine, accumulate in synovial tissues and fluids, mononuclear cells, and urine. Monocytes can hydrolyze the polyamines to ammonia and H_2_O_2_, which are cytotoxic and injure cells and tissues of the joint. Therefore, inhibition of transmethylation reactions and polyamine synthesis by MTX-PG are hypothesized to reduce synovial injury.^8^

In IBD, however, the evidence skews in the opposite direction. Polyamines may potentially be beneficial in IBD by promoting the renewal of IECs and enhancing intestinal barrier integrity via reinforcement of IEC junctions. Polyamines can also regulate the differentiation of monocytes into anti-inflammatory type macrophages within the lamina propria (reviewed by Nakamura and Matsumoto^41^). Given that MTX-PG inhibits transmethylation reactions and polyamine synthesis, the consequent perturbation of intestinal barrier homeostasis may contribute to the GI side effects associated with oral MTX administration.

#### 4 Nitric oxide synthase uncoupling

In addition to catalyzing the reduction of dihydrofolate to THF, DHFR also catalyzes the reduction of dihydrobiopterin (BH2) to tetrahydrobiopterin (BH4). BH4 is not only an antioxidant, but also an essential cofactor for NOS, a family of metabolic enzymes that can metabolize L-Arg and oxygen into nitric oxide (NO) and L-citrulline. BH4 binds at the interface between the 2 monomeric subunits of NOS, stabilizing the dimer and increasing L-Arg substrate interaction for NO production. Accordingly, decreased intracellular BH4 concentration promotes NOS destabilization and diminishes NO production. This dysfunction is described as uncoupling, as the reduction of oxygen is uncoupled from L-Arg hydroxylation and NO formation. Because electron transfer to oxygen is not inhibited, uncoupled NOS activity results in superoxide (O_2_) formation and a consequent increase in reactive oxygen species. BH2 can also bind to NOS with the same affinity as BH4 without cofactor activity, thereby competing with, and displacing, BH4 to inhibit NOS (reviewed by Gonçalves et al^42^). MTX, in its native glutamylated form, therefore, promotes NOS uncoupling to increase reactive oxygen species production (Fig. 2D). MTX-mediated reactive oxygen species production is linked to the induction of cell cycle arrest and apoptosis, as well as inhibition of NF-*κ*B-mediated proinflammatory signaling in T cells (discussed by Cronstein and Aune^8^). Conversely, NO, especially when generated by inducible NOS, is involved in inflammation, angiogenesis, and tissue degradation in RA (reviewed by Huang et al^43^). Similarly, it is well established that, although NO from inducible NOS is part of a prompt intestinal antibacterial response, it is also associated with the initiation and perpetuation of inflammation in IBD.^44^ In fact, inducible NOS expression and activity, and hence NO production, are generally increased in IBD, with more pronounced trends in UC compared with CD.^45^ Interestingly, NO generated by endothelial NOS appears to promote intestinal mucosal homeostasis via adequate perfusion and regulation of microvascular and epithelial permeability, suggesting NOS-specific effects.^44^ The therapeutic relevance of MTX-mediated NO inhibition, however, remains to be proven, both in RA and IBD.

#### 5 Novel pharmacodynamic properties

The biological landscape modulated by MTX continues to evolve, as evidenced by emerging data that demonstrate novel, and previously unreported, pharmacodynamic properties of this drug. Below we summarize these findings that are directly applicable to the gut and the treatment of IBD.

##### a T cells

**Fig. 3:**
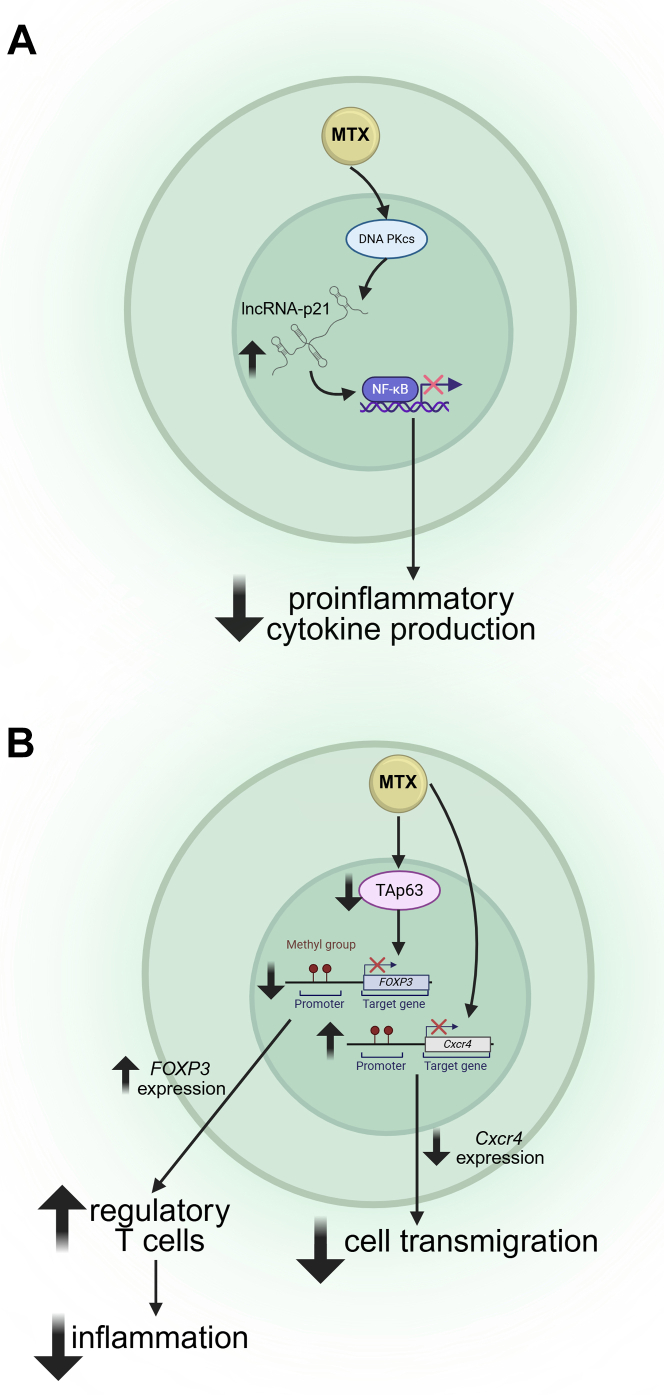
MTX alters T cell biology via transcriptional regulation and epigenetic modifications. (A) MTX induces expression of lncRNA-p21 in T cells through a DNA-PKcs-dependent fashion, resulting in decreased NF-*κ*B activity and expression of proinflammatory cytokines. (B) MTX downregulates TAp63, ultimately resulting in hypomethylation of enhancer site within the *FOXP3* locus, augmenting immune suppression by regulatory T cells with consequent reduction in inflammation. MTX also induces hypermethylation across the promoter region of *Cxcr4*, resulting in dampened CXCR4 expression and consequent decrease in cell transmigration. DNA PKcs, DNA-dependent protein kinase catalytic subunit; FOXP3, Forkhead box protein P3. Created in BioRender. Pizarro, T. (2025) https://BioRender.com/smmixvl.

The effects of MTX on T cells are increasingly complex and challenging to reconcile. Several studies indicate MTX may critically regulate gene expression through multiple mechanisms. For one, MTX regulates the expression and activity of noncoding RNA molecules, such as long (intergenic) noncoding RNA p21 (lncRNA-p21).^46^ LncRNA-p21 is a regulatory molecule known to alter transcription factor activity,^47^ with implications for modulating NF-*κ*B-mediated gene expression. In one study, patients with MTX-naïve RA are reported to express lower levels of lncRNA-p21 and greater NF-*κ*B activity compared with those patients treated with low-dose MTX. Mechanistically, MTX reduces NF-*κ*B activity by increasing lncRNA-p21 levels in T cells through a DNA-dependent protein kinase catalytic subunit-dependent mechanism (Fig. 3A).^46^ Although the relevance of lncRNA-p21 in IBD has not been reported to date, numerous lncRNAs are reported to promote inflammation, increase intestinal barrier permeability and dysfunction, enhance colonic cell apoptosis, pyroptosis, as well as autophagy, and suppress epithelial regeneration in patients with IBD, as well as in animal models of colitis (reviewed by Jiang et al^48^ and Heydari et al^49^). Given the contributions of lncRNAs to IBD pathophysiology, additional studies are needed to elucidate if and how MTX regulates lncRNAs in IBD.

Recent findings further support the notion that MTX’s effects on DNA and RNA biology extend beyond folate-dependent nucleic acid synthesis. For example, MTX was recently identified to downregulate transactivation domain containing tumor protein p63 (TAp63) in CD4^+^ T cells, which promotes hypermethylation at the enhancer site within the *FOXP3* (forkhead box protein P3) locus. The hypermethylation mediated by TAp63 results in suppression of *FOXP3*, which encodes a transcription factor that is critical for the development and function of regulatory T cells, consequently impacting immune suppression and exacerbating autoimmune arthritis. MTX, therefore, promotes the maintenance of regulatory T cells in autoimmune arthritis through TAp63 inhibition (Fig. 3B).^50^ MTX treatment also induces hypermethylation across the promoter region of *Cxcr4* (CXC-chemokine receptor 4), a chemokine receptor, with significant improvement in mouse models of arthritis.^51^ In fact, low-dose MTX is not only associated with downregulation of CXCR4 expression on peripheral T cells isolated from patients with RA, but also decreases in vitro cell transmigration (Fig. 3B). Together, these data indicate that MTX strongly modulates gene expression, at both epigenetic and transcriptional levels, with remarkable consequences to T cell biology.

##### b Goblet cells

**Fig. 4:**
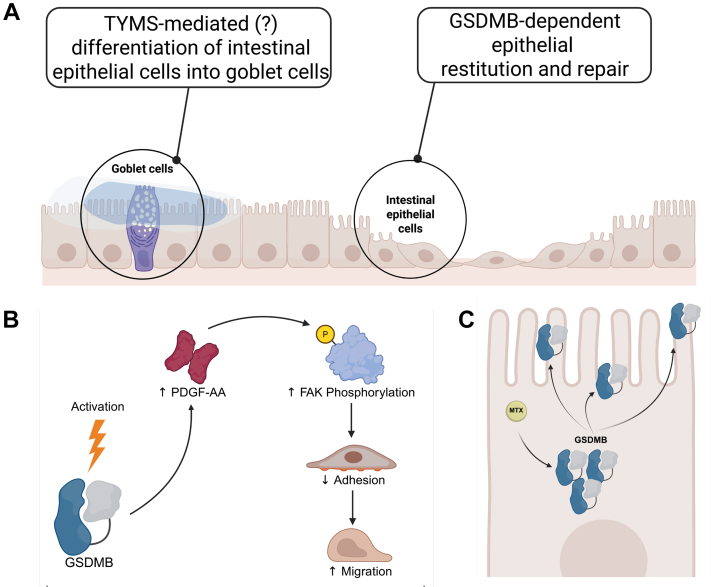
MTX alters IEC function. MTX confers differential effects on intestinal barrier function with alterations to epithelial cell differentiation, proliferation, migration, and adhesion. (A) MTX, potentially via TYMS resistance, promotes differentiation of IECs into mucus-secreting goblet cells, while inducing epithelial restitution/repair by regulating proliferation, migration and adhesion in a GSDMB-dependent manner. (B) GSDMB activation increases platelet derived growth factor A homodimer (PDGF-AA) levels, which subsequently induces focal adhesion kinase (FAK) phosphorylation, thereby altering cell adhesion dynamics to promote cell migration, a key component of intestinal epithelial restitution and repair. (C) MTX exposure increases GSDMB expression and its translocation to the plasma membrane in IECs. Figure created in BioRender. Pizarro, T. (2025) https://BioRender.com/smmixvl.

The effect(s) of MTX at the intestinal barrier differ greatly from those reported for RA. For one, it is well established within the IBD community that the IEC line, HT-29, (derived from colorectal adenocarcinoma cells) that develops resistance to MTX (ie, HT29-MTX) also develops a goblet cell-like phenotype, with enhanced secretion and synthesis of mucins, as well as expression of GI-specific mucin polypeptide epitopes.^52^^,^^53^ Moreover, HT29-MTX cells promote adhesion of both pathogenic and probiotic bacteria (ie, enterotoxigenic *Escherichia coli*,^54^ *Bifidobacteriaceae* species^55^), suggesting the potential impact of MTX on bacterial adhesion. This differentiation may involve the de novo pyrimidine synthesis pathway, as HT-29 cells resistant to 5-fluorouracil also differentiate into mucus-secreting cells.^53^ Given that 5-fluorouracil^56^ and MTX both inhibit TYMS once activated, additional studies are needed to elucidate how adaptation to TYMS inhibition may induce IEC differentiation into goblet cells (Fig. 4A).

##### c Intestinal epithelial cells

Recently, our group reported that gasdermin B (GSDMB) expression was elevated in IBD versus healthy patients.^57^ Functionally, GSDMB promotes effective intestinal wound healing (Fig. 4A), which is a key therapeutic goal in IBD management. GSDMB predominantly localizes to the intestinal epithelium in the GI tract and regulates IEC proliferation, migration, and adhesion. Mechanistically, GSDMB increases platelet derived growth factor A homodimer levels, which subsequently results in elevated focal adhesion kinase phosphorylation. Phospho-focal adhesion kinase alters cell adhesion dynamics, resulting in decreased adhesion and increased cell migration,^58^ thereby promoting IEC restitution (Fig. 4B).^57^ Curiously, MTX was identified as a small molecule that increases GSDMB expression in IECs. MTX exposure results in translocation of GSDMB to the plasma membrane, indicating MTX may also play a role in intracellular protein trafficking (Fig. 4C). Contrary to the established antiproliferative and antimigratory mechanisms mediated by MTX in other cell types, however, IECs show enhanced epithelial restitution and repair in a GSDMB-dependent fashion after exposure to MTX.^57^ Additional studies, however, are needed to parse out the underlying mechanism(s) mediating MTX-induced intestinal mucosal wound healing. The translational relevance of GSDMB during health and disease settings is currently unknown, however, these data suggest a novel role for MTX in promoting intestinal mucosal wound healing in a GSDMB-dependent fashion. The relevance of MTX on epithelial restitution is further corroborated by the fact that a single intravenous injection of MTX in rats results in an acute antiproliferative phase in intestinal crypts (evidenced by reduced mitotic bodies), followed by a highly proliferative phase (characterized by increased mitoses), with accelerated migration of enterocytes along villi.^59^ Although the in vivo effects may be attributed to a restitutive response following MTX-induced toxicity, the in vitro data from our group suggest that alternate mechanism(s) may be at play. It remains to be seen whether MTX can promote mucosal healing at the intestinal epithelial barrier in patients with IBD.

##### d Epithelial-to-mesenchymal transition

It is important to note that, in lung epithelial cells, such as A549 cells (derived from lung adenocarcinoma), MTX is reported to induce epithelial-to-mesenchymal transition (EMT) with enhanced migration, resulting in downregulation of E-cadherin and elevated levels of interleukin 6, *α*-smooth muscle actin, and transforming growth factor-*β*1.^60^, ^61^, ^62^ MTX-mediated EMT is likely folate-dependent, as folate supplementation inhibits EMT induced by conditioned medium from MTX treatment of lung epithelial cells.^62^ EMT is an embryonic program relaunched during wound healing and in pathological conditions, such as fibrosis.^63^ Given the clinical significance of wound healing and fibrosis in IBD, particularly in patients with CD, additional studies are needed to determine the relevance of MTX-mediated EMT in IECs.

##### e Gut microbiome

**Fig. 5:**
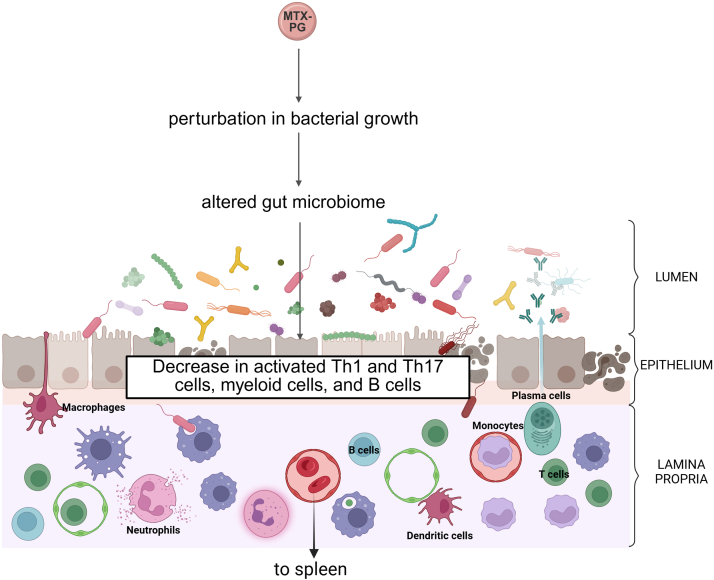
MTX alters the gut microbiome. MTX-PG perturbs bacterial growth via inhibition of purine/pyrimidine synthesis, resulting in altered gut microbiome composition (dysbiosis). MTX-mediated changes to bacterial flora results in decreased activation of gut mucosal and spleen T helper 1 (Th1)/T helper 17 (Th17), myeloid, and B, cells.

The gut microbiota has also recently emerged as a critical target of MTX, with important consequences on the host. MTX significantly reduces the *Bacteroidetes* phylum in germ-free mice colonized with stool samples from either healthy donors or patients with RA. As in mammalian cells, MTX also targets the purine and pyrimidine metabolic pathways in MTX-sensitive bacterial isolates, with subsequent perturbation in bacterial growth. Importantly, germ-free mice colonized with post-MTX stool samples of patients with RA display a reduction in activated T cells (T helper 1 and T helper 17 cells), myeloid cells, and B cells, in the spleen and/or intestinal mucosa during dextran sulfate sodium-induced colitis (Fig. 5).^64^ In mice with MTX-induced intestinal mucositis, *Bacteroidetes* is also dramatically altered, whereby gavage of mice with *Bacteroides fragilis* ameliorates MTX-induced inflammatory processes.^65^ Given the relevance of MTX in IBD, additional studies are needed to better elucidate the biological and clinical relevance of MTX’s impact on the gut microbiota.

### C Mechanism of resistance

**Fig. 6:**
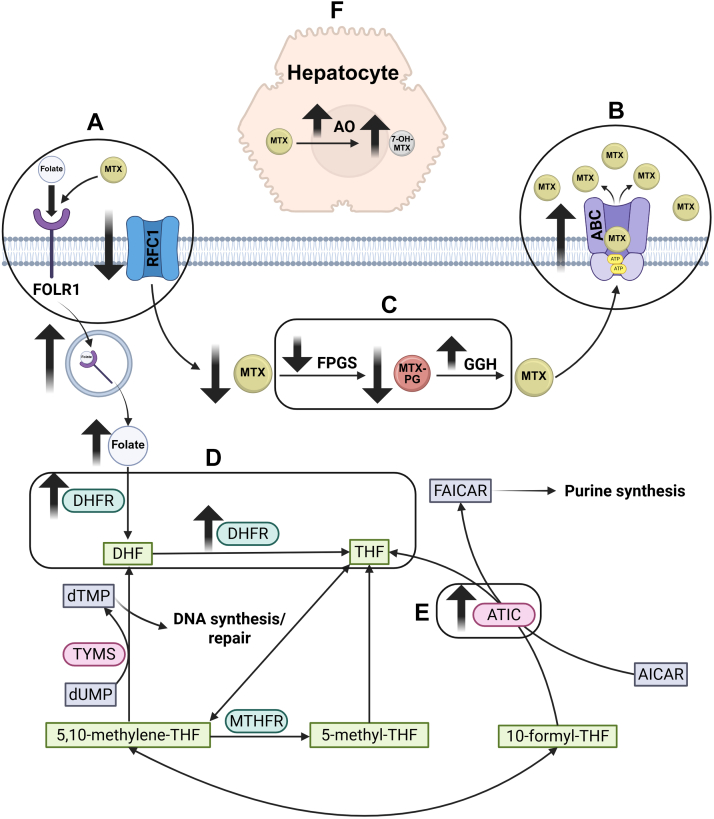
MTX resistance is mediated by changes in drug transport, metabolism, and folate-dependent pathways. (A) MTX influx is reduced by defective reduced folate carrier *α* (RFC1) activity and slow uptake by folate receptor 1 (FOLR1), which has lower affinity for MTX vs folic acid, whereas (B) MTX efflux is increased through various ABC transporters. (C) MTX polyglutamylation is impaired by reduced FPGS or increased GGH activity. (D) Increased DHFR activity/expression impairs MTX-mediated DHFR inhibition. (E) Increased ATIC activity impairs downstream MTX-mediated DHFR inhibition. (F) Enhanced hepatic metabolism to less active 7-OH-MTX results in decreased levels of MTX. DHF, dihydrofolate; dUMP, deoxyuridine monophosphate; FAICAR, 5-formamidoimidazole-4-carboxamide ribotide.

Several potential mechanisms of resistance have been identified for MTX. Although most of the evidence originates from the oncology literature, where higher doses are used, a few studies have examined MTX resistance in RA, which shares similar doses, although not route of administration, with IBD.^66^ Resistance mechanisms summarily affect MTX transport, metabolism, and folate-dependent pathways. Defective transport attributed to reduced folate carrier activity (via decreased protein levels or altered kinetics) and slow uptake via the folate receptor, which has a lower affinity for MTX than for folic acid, are thought to be involved (Fig. 6A). Increased drug efflux through ABC transporters, particularly P-glycoprotein, multidrug resistance-associated proteins, including ABCC1–ABCC5, and breast cancer resistance protein (BCRP/ABCG2), may further limit intracellular MTX accumulation (Fig. 6B), with polymorphic variations influencing transporter function. Interestingly, inhibition of some transporters is reported to improve drug concentration in preclinical models of RA.^67^ However, most associations have not been confirmed in independent cohorts.^68^^,^^69^ Other possible mechanisms of MTX resistance may be attributed to impaired polyglutamylation of MTX via reduced FPGS activity or increased GGH activity (Fig. 6C).^70^^,^^71^ Additionally, alterations in target enzymes, such as increased DHFR activity or expression, can kinetically alter DHFR (Fig. 6D), and enhance ATIC activity (Fig. 6E); in addition, increased folate and purine salvage pathways are linked to MTX resistance.^72^^,^^73^ Furthermore, hepatic metabolism of MTX to the less active 7-hydroxymethotrexate may also contribute to resistance (Fig. 6F).^66^ The clinical significance of these resistance mechanisms remains largely unclear; notably, none of these resistance mechanisms have been demonstrated in IBD.

The cumulative scientific evidence underscores the complex and pleiotropic pharmacology of low-dose MTX. Much of the current evidence on the effects of low-dose MTX on cellular biochemistry and overall cell biology, however, are limited to in vitro, ex vivo, and in vivo models of inflammatory arthritis, with information lagging as it applies to IBD. Such gaps in the current understanding of MTX pharmacology at the intestinal mucosal interface may contribute to the limited clinical use of MTX for IBD management. Indeed, it is highly likely that the target cell type, site-specific microenvironment, as well as the unique and complex etiologies underlying chronic inflammatory disorders, all contribute to MTX’s biological effects at the physiological level. However, the novel pharmacodynamic properties specific to the GI tract (namely goblet cells, IECs, and gut microbiota) warrant further exploration of MTX to achieve key therapeutic goals for IBD management. In the next 2 sections, we provide a comprehensive overview of current clinical evidence on the utility of low-dose MTX in IBD. We highlight current gaps in medical management and future perspectives to enhance MTX therapies for the treatment of IBD.

## III Crohn’s disease

### A Induction of remission

**Fig. 7:**
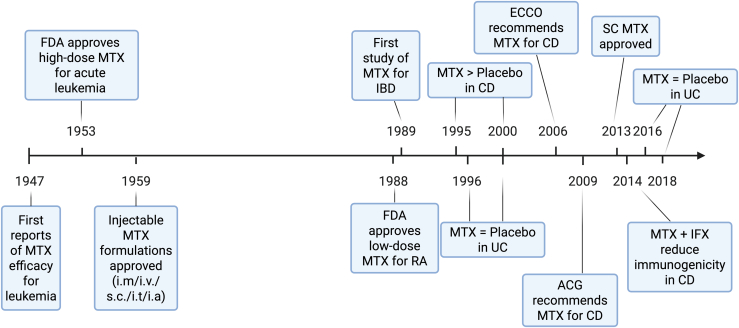
Timeline of MTX approval and clinical use in inflammatory and immune-mediated diseases. Key milestones in the regulatory approval and clinical applications of MTX, including use in leukemia, RA, and IBD. MTX efficacy was first reported for leukemia in 1947, with high-dose MTX approved by US Food and Drug Administration (FDA) for acute leukemia in 1953, followed by injectable formulations (intramuscular, intravenous, subcutaneous, intrathecal, and intra-arterial) in 1959. Low-dose MTX was approved for RA in 1988, whereas the first study in IBD was published in 1989, with randomized trial in 1995 demonstrating superiority over PBO in CD; subsequent trials showed no benefit over PBO in UC. Professional societies, including European Crohn’s and Colitis Organization (ECCO) and American College of Gastroenterology (ACG), recommended MTX for CD in 2006 and 2009, respectively. MTX (subcutaneous) was approved in 2013, and in 2018, combination therapy of MTX with IFX showed reduced immunogenicity in CD;. Created in BioRender. Pizarro, T. (2025) https://BioRender.com/smmixvl.

MTX has a long history of clinical use (common commercial names listed in Table 3) and was among the earliest medications investigated for the treatment of IBD. Its use as a therapeutic agent has spanned almost 80 years, with major milestones achieved during this period in regard to its discovery, regulatory approval, and therapeutic development (Fig. 7). Table 4 summarizes key US Food and Drug Administration-approved and other widely used indications for MTX. In regard to IBD, following the promising results of a small, noncontrolled study involving 14 CD and 7 patients with UC published in 1989,^74^ larger more rigorous clinical trials were initiated in the 1990s. Three randomized controlled trials subsequently assessed MTX for induction of remission in CD (summarized in Table 5),^75^, ^76^, ^77^, ^78^, ^79^, ^80^ and despite several meta-analyses attempting to pool together these results,^81^ the differences in route of MTX administration warrant individual consideration of each study. In fact, of the 3 trials, only 1 used parenteral MTX,^75^ whereas the other 2 examined lower doses of oral MTX. The trial assessing parenteral MTX^75^ randomized 141 patients with steroid-dependent to receive either 25 mg/wk i.m. MTX or placebo (PBO) for 16 weeks. By week 16, a significantly higher percentage of patients on MTX achieved symptomatic remission compared with those on PBO (39% vs 19%, relative risk: 2.06; 95% confidence interval: 1.09–3.89). Importantly, all participants, including those assigned to the PBO arm, were also taking prednisone, starting at 20 mg and tapering over 10 weeks, which may have reduced the observed effect size to the detriment of those assigned to the MTX group. Instead, the other 2 studies assessed efficacy for clinical remission of lower doses (12.5 or 15 mg, weekly) of oral MTX in patients with steroid-dependent CD and found only numerical, but not statistically significant, differences compared with PBO.^76^^,^^77^

**Table 3:** Examples of commercially available MTX formulations and routes of administration Limited to most commonly used; formulations may vary by region.

Brand Name	Route of Administration
Abitrexate	Oral/injection
Ebetrex	Oral/injection
Emthexate	Oral
Jylamvo	Oral solution
Lantarel	Oral/injection
Maxtrex	Oral
Metoject	Injection
Methoblastin	Oral/injection
Methofill	Injection
Nordimet	Injection
Otrexup	Injection
Rasuvo	Injection
Reditrex	Injection
Rheumatrex	Oral
Trexall	Oral
Xatmep	Oral solution
Zexate	Oral
Zlatal	Injection

**Table 4:** Clinical indications for MTX: FDA-approved and off-label use High-dose indications limited to oncology and differ significantly in route, dose, and monitoring requirements from low-dose MTX regimens used in immune-mediated diseases.

Condition	FDA-Approved	Details
Acute lymphoblastic leukemia	Yes	High dose
Non-Hodgkin’s lymphoma	Yes	High dose
Choriocarcinoma/trophoblastic neoplasms	Yes	High dose
Breast cancer, head/neck cancer, lung cancer, osteosarcoma	Yes	High dose
RA	Yes	Approved for severe, active RA; cornerstone of treatment
Polyarticular juvenile idiopathic arthritis	Yes	Approved for severe, active forms in children
Psoriasis	Yes	Approved when unresponsive to topical/systemic therapies
CD	No	Commonly used and supported by ECCO/ACG/AGA guidelines
UC	No	Occasionally used; efficacy inconsistent. Not formally recommended
Psoriatic arthritis	No	Widely used; supported by ACR/GRAPPA guidelines
Systemic lupus erythematosus	No	Used for arthritis and cutaneous symptoms
Ankylosing spondylitis/axial spondyloarthritis	No	Used for peripheral arthritis; no effect on axial disease
Dermatomyositis/polymyositis	No	Often used as a steroid-sparing agent
Granulomatosis with polyangiitis	No	Used in mild/moderate disease or maintenance
Microscopic polyangiitis	No	Similar to GPA; not for life-threatening disease
Sarcoidosis	No	Off-label use in pulmonary or extrapulmonary refractory cases
Atopic dermatitis	No	Rare off-label use in severe adult eczema
Autoimmune-associated interstitial lung disease	No	Used cautiously in selected cases

ACG, American College of Gastroenterology; ACR, American College of Rheumatology; AGA, American Gastroenterological Association; ECCO, European Crohn’s and Colitis Organization; FDA, US Food and Drug Administration; GRAPPA, Group for Research and Assessment of Psoriasis and Psoriatic Arthritis; GPA, granulomatosis with polyangiitis.

**Table 5:** Summary of randomized clinical trials using MTX for CD

First Author Year [Ref]	Dose (mg/wk)	Route	Endpoint	Population	Patients (*N*)	Study Arms	Treatment Duration	Concomitant Medications	Key Finding(s)
Feagan et al,^75^ 1995	25	Parenteral	Steroid-free clinical remission (CDAI < 150)	CDAI > 150, despite prednisone ≥12.5 mg	141	MTX vs PBO	16 wk	Tapered steroids	MTX superior to PBO
Arora et al,^76^ 1999	15–22.5	Oral	Clinical remission	Steroid-dependent	33	MTX or PBO	12 mo	Prednisolone ≥ 10 mg	No difference
Oren et al,^77^ 1997	12.5	Oral	Clinical remission	Steroid-dependent	84	MTX vs 6-MP vs PBO	9 mo	Steroids and 5-ASA	No difference
Feagan et al,^78^ 2014	10–25	Parenteral	Steroid-free clinical remission	Active CD, initiated on prednisone induction	126	IFX + MTX vs IFX	50 wk	Tapered steroids	No difference
Feagan et al,^79^ 2000	15	Parenteral	Worsening of symptoms (>100 CDAI)	Patients in symptomatic remission (CDAI < 150) after MTX induction	76	MTX vs PBO	40 wk	None	MTX superior to PBO
Schröder et al,^80^ 2006	20	Parenteral, then oral	Clinical remission (CDAI < 150)	Active CD, failed AZA	19	IFX + MTX vs IFX	48 wk	Steroids, 5-ASA	No difference

5-ASA, 5-aminosalicylic acid; CDAI, Crohn’s disease activity index.

### B Maintenance of remission

The evidence supporting the use of parenteral MTX as a maintenance therapy comes from a double-blind, randomized, PBO-controlled trial. In this study, patients with steroid-dependent CD, who previously achieved remission after 16 to 24 weeks of treatment with 25 mg i.m. MTX once a week, were randomized to receive either 15 mg i.m. MTX once weekly or PBO for 40 weeks. Steroids, or other additional treatments for CD, were allowed during the study period. At week 40, a higher percentage of patients in the MTX group remained in clinical remission compared with the PBO group (65% vs 39%, relative risk: 1.67; 95% confidence interval: 1.05–2.67).^79^ Interestingly, in a small subgroup of patients, who relapsed during the maintenance phase, remission was regained by increasing MTX’s dose back to 25 mg/wk, as in the induction phase, in combination with a steroid course. These data support the use of a higher dose of MTX for maintenance in patients with suboptimal control on 15 mg MTX. A second randomized PBO-controlled trial assessed oral MTX, but failed to observe differences in either maintenance or induction,^77^ as mentioned previously in the induction section. Finally, a small study comparing 25 mg/weekly MTX (administered intravenous during induction and orally during maintenance) with azathioprine (AZA) found similar clinical remission rates between the 2. Although neither of the differences were statistically significant, remission rates were numerically higher at 3 months for MTX (44% MTX vs 33% AZA), whereas at 6 months for AZA (56% MTX vs 63% AZA), suggesting that the switch to the oral formulation of MTX reduced the therapeutic benefits.^82^

Based on these studies, the recommended formulations of MTX for use in IBD are parenteral, either intramuscularly or—because the 2 are bioequivalent—subcutaneously. Because subcutaneous injections are less painful, this is the preferred choice for MTX administration in clinical practice. Importantly, none of the prospective PBO-controlled studies have assessed mucosal healing or endoscopic endpoints, resulting in a significant knowledge gap regarding the efficacy of MTX on more objective measures of response. This is reflected in scientific guidelines. The American College of Gastroenterology, until recently, conditionally recommended, with limited evidence, that MTX up to 25 mg i.m. or s.c. once weekly should be considered for alleviating signs and symptoms in patients with steroid-dependent CD and for maintaining remission.^83^ The latest update of the guidelines in 2025^84^ aligned with those from the American Gastroenterology Association and the European Crohn’s and Colitis Organization, removing mention of “alleviation of signs and symptoms” and steroid-dependency, and increasing the quality of evidence to “moderate.”^2^^,^^85^

### C Combination approaches with methotrexate

After the landmark SONIC (Study of Biologic and Immunomodulator Naïve Patients in CD) study, demonstrating the superiority of AZA plus IFX compared with either drug alone,^86^ the COMMIT (Combination MTX and IFX for CD Treatment) study was designed to test combination MTX (starting at 10 mg/wk escalated to 25 mg/wk, s.c.) plus IFX versus IFX monotherapy^78^; disappointingly, the study found no significant difference between the 2 arms. These results were unexpected, not only because of the prior results from the SONIC trial, but also in light of the well established synergistic effect of MTX with anti-TNFs in RA.^87^^,^^88^ In hindsight, possible flaws are hypothesized in the broad patient selection. The primary endpoint was symptomatic remission, although no minimum symptomatic activity was required at inclusion, and nearly 30% of patients in both arms had a CD Activity Index score of <150 (indicating remission) at baseline, meaning they were unlikely to improve, regardless of treatment. Additionally, no baseline endoscopy was performed, which may have allowed the inclusion of patients without active disease, further diluting the observed effect size.^89^ Despite methodological criticisms of the COMMIT trial, MTX’s effects on mucosal healing have also been disappointing in another prospective study that compared MTX, AZA, and IFX in achieving ulcer-free endoscopy and found significantly lower rates of endoscopic healing in the MTX-treated group compared with those receiving AZA or IFX.^90^

Nevertheless, the COMMIT study did observe a clear effect of MTX on anti-IFX antibody formation (4% in the combination arm versus 20% in the monotherapy arm) and IFX trough levels (6.35 vs 3.75 *μ*g/mL). This aligns with earlier findings that both AZA and MTX are effective in reducing IFX immunogenicity.^91^ A synergistic effect of MTX on IFX appears to stem from reduced immunogenicity, translating to fewer secondary failures and adverse events; however, the relatively short duration of the COMMIT trial may have failed to fully capture these benefits. MTX and AZA appear to be similarly effective in preventing immunogenicity and maintain anti-TNF levels; nevertheless, anti-TNF combination with AZA results in superior remission rates^92^ and therefore, is typically preferred, whereas MTX is mainly used when AZA is not recommended (eg, Epstein-Barr negative serology, thiopurine intolerance).^93^

Further reinforcing the notion that MTX acts through multiple mechanisms is the observation that, when used in combination with IFX, lower doses of MTX than those used in monotherapy appear to be sufficient to prevent immunogenicity. A retrospective study of 163 patients found no significant difference in efficacy between high-dose (>12.5 mg/wk) and low-dose (≤12.5 mg/wk) MTX for inducing, or maintaining, remission with anti-TNF agents.^94^ Even more so, one study found no difference in immunogenicity between doses, including for oral MTX.^95^ Thus, MTX is recommended as an adjunct therapy to reduce immunogenicity of concomitant biologic therapies.^84^

## IV Ulcerative colitis

Data on MTX efficacy in UC are more conflicting than those in CD (summarized in Table 6).^96^, ^97^, ^98^, ^99^ In the first noncontrolled report by Kozarek et al.,^74^ 5 out of 7 (71%) patients with UC, who received 25 mg/weekly i.m. MTX for 12 weeks followed by oral MTX maintenance, achieved a symptomatic response and histologic improvement; however, none reached the desired outcome of mucosal healing. This motivated more rigorous investigation of MTX for UC. Indeed a few years later, the first randomized PBO-controlled trial was conducted in Israel comparing oral 12.5 mg/weekly MTX versus PBO in patients with steroid-dependent UC.^96^ Unfortunately, the study failed to observe any statistically significant difference in monthly steroid use, symptoms scores, or endoscopic measures of inflammation. Around the same time, another small (*N* = 26) randomized, controlled, study compared the addition of oral MTX to mesalamine versus mesalamine alone and did not observe benefits in either symptoms, endoscopy or histology.^98^ These disappointing results paused further investigation of MTX in UC. However, because more retrospective observations continued to show positive results,^100^^,^^101^ 2 randomized PBO-controlled trials of parenteral 25 mg/weekly MTX were designed, 1 in Europe and 1 in the United States. The European METEOR (controlled, randomized, double-blind, Multicenter Study Comparing MTX versus PBO in Steroid Dependent UC) study did find significantly higher clinical remission rates (42% vs 24%, *P* = .04) among MTX-treated patients. However, differences were not confirmed for steroid-free remission and endoscopic remission (20% vs 32%, *P* = .15 and 35% vs 25%, *P* = .28, respectively), suggesting that the benefits may have come from concomitant steroids.^97^ The American study by Herfarth et al^99^ compared parenteral MTX versus PBO in the maintenance of remission after response to MTX induction. Patients with steroid-dependent UC were treated with MTX for 16 weeks, after which 51% went into remission; those in remission were randomized to either continue MTX for maintenance or switch to PBO until week 48. At the end of the follow up, there was no difference in relapse rates (60% in the PBO group vs 66% in the MTX group, *P* = .75) nor in steroid-free clinical remission (*P* = .86).^99^ Based on these studies, both European and American guidelines do not recommend use of MTX in UC, for either induction, or maintenance, of remission.^2^^,^^3^ However, it is worth noting that MTX combined with biologics has not been rigorously investigated in UC, unlike for CD, leaving an important knowledge gap. In this regard, it is worth considering that MTX efficacy in preventing immunogenicity is not specific to a type of IBD, and therefore MTX could still be beneficial, in the context of UC, by increasing the duration of other biologics, particularly IFX.

**Table 6:** Summary of randomized clinical trials using MTX for UC

First Author Year, [Ref]	Dose (mg/wk)	Route	Endpoint	Population	Patients (*N*)	Study Arms	Treatment Duration	Concomitant Medications	Key Finding(s)
Oren et al,^96^ 1996	12.5	Oral	Clinical remission	Mayo ≥ 7	67	MTX vs PBO	9 mo	Steroids, 5-ASA	No difference
Carbonnel et al,^97^ 2016	25	Parenteral	Steroid-free remission (including endoscopy); steroid-free clinical remission	Steroid-dependent	111	MTX vs PBO	24 wk	Tapered steroids	No difference in steroid-free remission, but greater symptomatic remission
Onuk et al,^98^ 1996	15	Oral	Change in symptoms, endoscopy and histology	N/A	26	MTX + 5-ASA vs MTX	12 mo	5-ASA	No difference
Herfarth et al,^99^ 2018	25	Parenteral	Relapse free; combined clinical/endoscopic response	Responded at MTX induction	84	MTX vs PBO	32 wk	5-ASA	No difference

5-ASA, 5-aminosalicyclic acid; N/A, not applicable.

## V Safety data

### A Adverse events

Consistent with the scope of this review, we discuss safety concerns related to low-dose MTX, as used in IBD and other inflammatory conditions. The overall tolerability of low-dose MTX is a matter of debate. Adverse events are reported in a significant proportion of patients ranging from 10% to 40%, with the most common complaints being GI, such as nausea and vomiting, or nonspecific general malaise, headache, and low-grade fever.^102^ However, despite their frequency, these are mostly mild, self-resolving, and typically present soon after injection. Nevertheless, adverse events lead to treatment discontinuation in a considerable number of patients, and at higher rates compared with thiopurines.^103^

MTX is also associated with mild and transient alterations in liver function tests in approximately 10% of cases,^104^ and in rare instances, with more concerning liver fibrosis. The latter develops soon after MTX is started, and is associated with high-dose MTX, but not with the duration of treatment and cumulative dose received; it is more often observed in patients with pre-existing liver conditions.^105^^,^^106^ Combining folic acid with MTX is a well established practice in RA to reduce MTX-related GI side effects and liver toxicity. Data on folic acid supplementation specific to the setting of MTX for the treatment of IBD are limited, but in one study, patients not receiving concurrent folic acid supplementation were 5 times more likely to experience side effects compared with those taking at least 5 mg weekly.^107^ In a recent systematic review of 12 studies reporting folic acid combination with MTX for IBD, various administration patterns were used without a clear standard.^93^ Therefore, for the duration of MTX therapy, a minimum of 5 mg of folic acid per week is recommended, to be administered on a day different from that of MTX (eg, 2–3 days after MTX or in 1 mg daily dose for 5 days each week).^108^

Other than liver fibrosis, another rare and severe adverse event associated with MTX use is pulmonary fibrosis. The incidence of lung fibrosis due to MTX is debatable, partially because of the difficulty in separating the risk of pulmonary fibrosis associated with RA (the main indication for low-dose MTX) from that of MTX itself. A systematic review of 3463 patients with RA receiving MTX reported 84 cases of pulmonary toxicity, of which 15 (0.43%) were attributed to MTX.^109^ Although 2 relatively specific complications of MTX involve fibrosis development in liver and lung, there is no data suggesting an increased risk of fibrostenotic complications of CD, which occur in up to 50% of patients with CD. Speculatively, this may reflect tissue-specific effects of MTX, differences in dosing, or the influence of active inflammation, an element not typically central to MTX-induced liver or lung fibrosis, and instead invariably present at the site of intestinal CD-associated fibrosis.

The infection risk associated with MTX is relatively modest and broadly lower than that of most biologics. Indeed, a retrospective study of 100 patients with IBD found no increase in the risk of opportunistic infections in patients treated MTX,^110^ whereas other studies confirmed the safety profile in the elderly compared with younger patients.^111^ For these reasons, MTX is considered a safe treatment option for patients with a high risk of opportunistic infections. Similarly, the risk of malignancies is reassuring and likely lower than that for thiopurines, although data are limited.^112^^,^^113^ In this regard, because lymphomas have been associated with prolonged exposure to AZA and 6-mercaptopurine,^114^ and are typically observed in patients with negative Epstein-Barr serology, such as in children, MTX is increasingly preferred over thiopurines in pediatric patients with IBD.^115^

Finally, maternal exposure to MTX is highly teratogenic and associated with several congenital anomalies and spontaneous abortion.^116^ In addition, it is also excreted in breast milk, thus exposing newborns to possible immunosuppression. For these reasons, MTX is contraindicated for women who desire to conceive, and strictly contraindicated for pregnant and breastfeeding women. Conversely, paternal exposure to MTX does not increase the risk of spontaneous abortion and major birth defects^117^^,^^118^; however, it does reduce sperm count, although it is unclear whether this has any clinical implications.^119^ Altogether, teratogenicity is the main clinical aspect of MTX with a notable sex assigned at birth-related difference.

Although not observed in IBD, when MTX is used as a chemotherapeutic agent at high doses, the safety profile changes considerably with nephro-, neuro-, and hematological toxicities as the primary causes of concern. In addition, rare idiosyncratic reactions manifesting as cutaneous rashes have been described, including severe ones, such as those found in Stevens–Johnson syndrome. The main safety concerns related to low-dose MTX are summarized in Table 7.

**Table 7:** Summary of common and clinically relevant adverse events/toxicities associated with low-dose MTX use

Adverse Event/Concern	Frequency	Details
GI intolerance	Common (20%–30%)	Most commonly nausea, abdominal discomfort; improved with s.c. route and folate
Fatigue/malaise	Common (10%–20%)	Often transient and dose-related
Elevated liver enzymes	Common (15%–30%)	Typically mild; persistent elevation may require dose adjustment
Mild cytopenia	Uncommon (∼5%)	More likely with renal impairment or drug interactions
Stomatitis/oral ulcers	Uncommon (1%–5%)	Preventable with folic acid supplementation
Alopecia/skin rash	Uncommon (1%–3%)	Mild and reversible
Mild infections	Uncommon	Slight increased risk, especially with combination therapy; likely lower than anti-TNF agents
MTX-induced pneumonitis	Rare (<1%)	Idiosyncratic; presents with dyspnea, cough, fever
Liver fibrosis/cirrhosis	Rare (<1%)	Risk increases with alcohol, obesity, persistent elevation in LFT
Severe myelosuppression	Rare (<1%)	Linked to renal failure, overdose, or interacting drugs (eg, TMP-SMX)
Teratogenicity	High/always relevant	Contraindicated in pregnancy; contraception required for both sexes
Renal clearance–related toxicity	Very rare at low dose	Monitor renal function, especially in elderly
Folate deficiency	Common without folate	Supplementation (1–5 mg/wk) reduces GI and hematologic toxicity

LFT, liver function test; s.c., subcutaneous; TMP-SMX, trimethoprim-sulfamethoazole (antibiotic used to treat various bacterial infections).

### B Drug interactions

MTX is subject to several drug interactions, which are primarily observed at high doses, but also possible at the lower doses used in inflammatory conditions. Because more than 80% of MTX is eliminated through urine excretion via glomerular filtration and tubular secretion, drugs that impair these processes can increase circulating MTX levels and lead to toxicity. Nonsteroidal anti-inflammatory drugs, which are known to reduce glomerular filtration rate, are a particular focus in this context. Given their widespread use in patients with RA, several studies have investigated the potential interaction(s) between nonsteroidal anti-inflammatory drugs and MTX.^120^, ^121^, ^122^, ^123^, ^124^, ^125^ The clinical significance of this interaction, however, appears to be modest at doses used for inflammatory diseases. Moreover, in the setting of IBD, nonsteroidal anti-inflammatory drugs are far less commonly prescribed because of their potential to exacerbate intestinal inflammation, making this interaction less relevant in clinical practice. Other drugs that may increase MTX serum levels that interfere with MTX renal clearance are probenecid, proton pump inhibitors (PPIs), and penicillin. Probenecid inhibits renal tubular transporters responsible for MTX secretion,^126^ but the exact mechanism by which PPIs increase MTX levels is uncertain.^127^ Two hypotheses, both involving renal excretion, have been proposed. The first suggests that PPIs also inhibit the H^+^/K^+^-ATPase in renal epithelial cells, reducing active tubular secretion of MTX.^128^ However, studies showing no change in urinary pH after omeprazole use cast doubt on the clinical significance of this pathway.^129^ The second hypothesis involves PPI-mediated inhibition of the breast cancer resistance protein, an efflux transporter for MTX in the proximal tubule, although to reach the effective inhibitory concentrations, standard PPI doses would not be sufficient.^130^ Finally, penicillin and some cephalosporins compete for renal tubular secretion, potentially reducing MTX clearance.^131^ Overall, at low doses, MTX’s interactions related to renal excretion are rare and should not pose a significant barrier to its use.

In contrast to the aforementioned interactions, toxicity from cumulative antifolate use can occur with trimethoprim, a bacteriostatic antibiotic that selectively inhibits bacterial DHFR, but at higher doses or with prolonged use, can partially inhibit its usual potentiating effects of MTX. The combination of MTX and trimethoprim can lead to severe, possibly life-threatening, toxicity. Although this interaction is well recognized in oncology due to the higher doses used, there may be less awareness among gastroenterologists, who are typically less familiar with these agents.^132^ Another potential cause of toxicity is the displacement of MTX from albumin-binding sites by drugs, such as sulfonamides or tetracyclines, particularly in the setting of low serum albumin concentrations. Additionally, drugs that interfere with hepatic metabolism, such as some anticonvulsants, may also alter MTX PKs. Altogether, although several interactions have been described, almost all are primarily associated with high-dose MTX. Conversely, low-dose MTX should be considered a relatively safe drug. As such, although numerous drug interactions with MTX have been reported, the vast majority are clinically significant only at high-dose regimens. At the low doses typically used for inflammatory conditions, MTX should be considered a relatively safe drug. However, maintaining awareness of potential drug–drug interactions remains important to prevent avoidable toxicity, even in this setting.^109^^,^^133^^,^^134^

## VI Future directions

### A Pharmacogenomics

Multiple genetic variants are associated with alterations in function of proteins involved in MTX transport or intracellular metabolism. At least 13 mutations in *DHFR* are associated with differences in either toxicity or efficacy of MTX.^135^ However, none of the associations have been verified in independent cohorts. In addition, genetic variants affecting the metabolism of high-dose MTX might not be clinically relevant for the low-dose MTX used in inflammatory conditions, including IBD.^136^ In the context of inflammation, the most consistently studied polymorphism is the TT genotype of *MTHFR* C677T (rs1801133) that leads to a reduction of MTHFR enzyme activity, resulting in increased levels of homocysteine, which can be toxic. Therefore, it has been proposed that patients with the C677T variant may benefit from a dose reduction, although this has not been prospectively tested.^137^ Conversely, adult patients with RA, who possess the A allele of *SLC19A1* G80A (rs1051266), may experience a better response to MTX due to improved substrate binding and/or translocation.^138^^,^^139^ Finally, patients with RA carrying the 3R allele of *TYMS* 28 bp VNTR (rs34743033) might require a higher initial dose of MTX because the 3R allele, located in the 5′-UTR region of *TYMS*, promotes mRNA trancript expression and downstream enzymatic (protein) activity. *Because* TYMS is a target of MTX, an increased number of TYMS molecules may necessitate a higher MTX dose to achieve therapeutic effectiveness.^140^ Several genetic variants, including *SLCO1B1*, *ABCC2*, *ABCB1*, ABCC4, *FPGS*, and *GGH*, are also reported to be linked to MTX response and/or adverse events; although, unfortunately, none of these have been confirmed in independent cohorts.

In the setting of IBD, where MTX’s safety concerns are relatively modest, the main rationale for genetic testing would be to identify patients who are more likely to benefit from treatment. However, as MTX response is influenced by multiple genes, each with a small effect, testing a single polymorphism is unlikely to yield great clinical value. In addition, as genes may interact with each other, linkage disequilibrium should be taken into consideration.^141^

### B Clinical perspectives

MTX is an important drug in the armamentarium to treat patients living with CD. However, it remains underutilized compared with its use in other immune-mediated conditions, partially because of misconceptions in regard to safety and patient tolerance.^107^ Biologics and novel small molecules are generally more effective than conventional antimetabolites, such as MTX, and in general, have progressively overshadowed them. However, new therapies are also considerably more expensive, and their cost-effectiveness is based on Western pricing, and may not apply to resource-limited settings. In this context, an affordable drug, such as MTX, can be particularly valuable. It is therefore important that MTX remains recognized in treatment guidelines and incorporated into educational initiatives tailored to economically constrained environments. Although the results of controlled studies using MTX for the treatment UC have not demonstrated convincing benefits, it is plausible that MTX retains a modest efficacy. Although this is not sufficient to justify MTX use as a single agent, it is still worth considering as a possible addition to other drugs when UC cannot be effectively controlled and when other medications are not available.

Nevertheless, in countries where biologics and targeted small molecules are also more readily available and cost does not necessarily play a paramount role, MTX remains an important option. A recent study from the Spanish national registry ENEIDA (Estudio Nacional en Enfermedad Inflamatoria Intestinal Sobre Determinantes Genéticos y Ambientales) challenged the common perception of MTX as a second-rate drug, showing that MTX could induce symptomatic response and remission in 60% and 30%, respectively, of patients with CD who previously failed one (39%) or more (61%) anti-TNF agents.^142^

An important consideration supporting the use of MTX in CD is the limited range of therapeutic options available for patients with mild to moderate disease activity. While in UC, 5-amino salicylic acid (such as mesalamine) remains a safe, effective, practical, and affordable first-line therapy for mild to moderate disease, no equivalent option exists for CD. In cases of moderate to severe CD, biologics and advanced small molecules are the recommended first-line treatments, but the management of mild to moderate CD remains a therapeutic gray area, with relatively few options available. In this setting, thiopurines (AZA and 6-mercaptopurine) and MTX are the only conventional immunomodulators formally approved for use. These drugs continue to have an important role and should be considered over leaving patients untreated or relying on recurrent courses of corticosteroids, which carry well known risks and fail to achieve sustained control of disease. Notably, prolonged exposure to AZA has been associated with an increased risk of lymphoproliferative disorders, raising concerns over its long-term safety. In contrast, low-dose MTX carries fewer cumulative exposure-related risks, making it a suitable candidate for sustained disease management in appropriate patients.

MTX is also well suited for combination therapy with advanced drugs, other than IFX, for which it is commonly added to reduce immunogenicity. In fact, considering that a significant proportion of patients do not respond to treatment, or lose response over time, the need for enhanced therapeutic strategies remains high. The combination of different therapies has attracted interest and yielded some promising initial results. However, such strategies are limited by high costs and safety concerns, particularly related to immunosuppression. In this context, MTX could represent a cost-effective and relatively safe add-on treatment for multirefractory patients. The pioneering EXPLORER study recently showed that MTX can be safely and effectively combined with adalimumab and vedolizumab, 2 monoclonal antibodies targeting TNF and *α*4*β*7 integrin, respectively, in patients with recently diagnosed, high-risk CD.^143^ Although the study was small and uncontrolled, the promising symptomatic and endoscopic remission rates (54.5% and 34.5% at 6 months, respectively) support further investigation of MTX in combination with other advanced therapies.

The lack of efficacy of oral formulations and the need for subcutaneous or intramuscular injections have been major obstacles for the widespread use of MTX. Indeed, real-world data confirm that oral AZA is usually preferred as a first-choice immunomodulator.^144^ Interestingly, however, in RA, one study showed that splitting a 25 to 30 mg oral MTX dose into 2 administrations taken 8 hours apart significantly increases its bioavailability.^145^ Similar studies in IBD have not yet been conducted, but would be highly valuable, as they may re-establish oral MTX as a viable and more practical therapeutic option.

The other main limitation to MTX’s use has been safety concerns, especially in the context of IBD, for which other effective options are available. AZA, the main “competitor” of MTX in IBD, is also burdened by considerable adverse events, but unlike MTX, some of these can be prevented by testing for enzymatic activity before starting treatment; unfortunately, the same is not possible for MTX. However, a recent study in CD, and previous ones in RA, suggest that the concentration of MTX-PG (Fig. 1B) in RBCs, particularly MTX-PG_3_, positively correlates with efficacy and inversely with GI adverse effects.^145^^,^^146^ If confirmed in other studies, early testing of MTX-PG_3_ could inform dose adjustments, with the end goal of reducing toxicity.

Finally, extraintestinal manifestations are common in patients with IBD, with prevalence varying from 6% to 47%, depending on the definitions,^147^^,^^148^ as well as in other immune-mediated inflammatory conditions. Because MTX is effective for the treatment of several such conditions, its use can be leveraged to achieve a combined effect to treat IBD and other immune-mediated conditions, or their extraintestinal manifestations, whereas the same is not possible with thiopurines. In particular, MTX is effective in peripheral spondyloarthropathy and RA, as well as in psoriasis, scleritis, and certain types of uveitis.^93^

### C Advances in nanomedicine

In recent years, advances in nanomedicine have resulted in re-evaluating the drug delivery platform for various pharmacologic agents. MTX is no exception. Nanoparticle-based MTX delivery carries great potential, as it may enhance its therapeutic efficacy via targeted delivery, while simultaneously reducing off-target side effects. In one study, MTX-loaded yeast glucan particles were observed to undergo effective phagocytosis by murine macrophages (through dectin-1 and CR3 receptors), while effectively suppressing the proliferation of macrophages. Such targeted reductions in macrophage number results in downregulation of proinflammatory cytokines upon stimulation with lipopolysaccharide. Importantly, MTX-yeast glucan particles was shown to accumulate at sites of inflammation in a murine model of experimental colitis, suggesting targeted drug delivery.^149^ However, the use of yeast glucan particles may induce off-target side effects as yeast glucans have immunogenic and immunomodulatory properties that extend beyond macrophage function. Recently, nanoparticles with colonic pH-sensitive drug release properties were tested for targeted MTX delivery. The MTX-loaded nanoparticles, named MTX@hCEP, similarly enhanced cellular uptake of MTX by murine macrophages, thereby inhibiting proliferation and secretion of proinflammatory cytokines upon liposaccharide stimulation. Importantly, MTX@hCEP not only accumulates in the colons of experimental colitic mice, but shows extended retention time of MTX in the colon. Moreover, MTX@hCEP enhances in vivo mucosal repair, in addition to alleviating inflammatory symptoms.^150^ Alternatively, nanocarriers can be leveraged to not only enhance targeted drug delivery, but can also allow coloading of chemically incompatible drugs for combination therapy. For instance, niosomes (nanovesicles) loaded with silibinin, a hydrophobic drug, and MTX, a hydrophilic drug, effectively increases the potency of this combination therapy (IC_50_ = 2.6 *μ*g/mL via niosome delivery vs 6.85 *μ*g/mL silibinin-MTX alone), while promoting in vitro apoptosis in colon cancer cells.^151^ This technology can also be used to overcome multidrug resistance with enhanced combination therapy. Its therapeutic potential is further supported by the fact that codelivery of metformin, an antidiabetic agent identified to hinder metastasis and trigger apoptosis,^152^ with MTX via nanoparticles enhances inhibition of tumor growth in a xenograft mouse model of triple-negative breast cancer.^153^ Other innovations in nanomedicine focus on nanomaterials, from synthetic polymers to metal organic frameworks and biocomposite beads, to enhance MTX delivery primarily to target cancer cells.^154^, ^155^, ^156^ Although altogether promising, most studies on nanoparticle-mediated MTX delivery are predominantly limited to in vitro cell line models, with the primary focus on therapeutic development of antineoplastic agents. Given the clinical importance of MTX in chronic inflammatory diseases, such as IBD, additional studies are needed to investigate the clinical potential of MTX-based nanomedicine for IBD management.

### D Combatting drug resistance

A novel approach to mitigate MTX resistance leverages targeted proteolysis. Specifically, proteolysis targeting chimeras hijack cellular quality control machinery to catalytically and selectively degrade proteins of interest (via proteasome- and E3 ligase-dependent activity). Proteolysis targeting chimeras (PROTACs) offer alternative approaches to drugging DHFR, MTHFR, ATIC, TYMS, and other potential MTX targets. Versortrexate,^157^ a potent MTX-PROTAC analog, effectively induces selective DHFR-degrading activity accompanied by low cellular toxicity. Although it is currently categorized as a chemical tool to study the pharmacology of antifolate therapeutics, development of MTX-PROTACs may open the door to proteolysis, as a novel mechanism to target folate-dependent enzymes, and to overcome MTX resistance.

### E Leveraging novel mechanisms of action

The current literature on MTX’s pharmacology, clinical efficacy, and therapeutic innovation is heavily skewed toward targeting the immune compartment, especially in patients with RA. However, given the clinical relevance of low-dose MTX in IBD management, additional studies are needed to identify gaps in our understanding of MTX pharmacology in the GI tract. The intestinal mucosal barrier and surrounding gut microbiota are critically affected by MTX, with differential modulation of bacterial cell growth, as well as epithelial cell differentiation, proliferation, migration, and adhesion. These effects can subsequently impact the mucosal immune system, thereby altering not only the therapeutic responses for IBD, but also the GI-associated toxicities often reported by patients. Although several mechanism(s) of action regarding the therapeutic effects of MTX have been proposed for IBD, additional research is necessary, particularly regarding various gut mucosal cell populations and IBD-specific immunopathology. Precise identification of MTX’s apparent wide-range of effects, for example on intestinal mucosal wound healing, will provide new insights that can inform and refine MTX-based therapies for IBD.

## VII Conclusions

MTX remains an important therapeutic option for CD, however, in UC, MTX’s benefits are insufficient to justify its use as a monotherapy. Nonetheless, it remains worth exploring how more targeted delivery methods or combination approaches with other drugs could enhance its efficacy in the latter setting. In addition, MTX is highly effective at reducing the immunogenicity of other drugs, both in CD and UC. Importantly, emerging data suggest an additional role in shaping the gut microbiome, as well as mucosal healing, beyond its well established anti-inflammatory effects, particularly targeting immune cell populations. Given the evident differential efficacy of MTX in IBD compared with RA, it is likely that current therapeutic regimens do not consider MTX’s mechanism(s) of action unique and specific to the intestinal mucosal barrier and/or IBD-specific immunopathology. The dose and regimen required for this effect, in order to become clinically meaningful, remains uncertain. Although scattered evidence from oncology and RA has identified potential mechanisms of MTX resistance, their relevance in IBD remains largely unexplored. Translating these findings into clinically actionable insights is essential to optimize MTX use and identify patients who may benefit most. Future research should focus on elucidating resistance mechanisms, including the impact of genetic polymorphisms on MTX metabolism and efficacy, and integrating this knowledge into individualized treatment strategies. Additionally, efforts should be directed at defining MTX’s role at the intestinal mucosal barrier, which can inform, and perhaps improve, therapeutic outcomes. Moreover, such insights can aid in refining combination regimens with biologics or small molecules to strengthen existing therapeutic options to treat patients with IBD. Advancing research across these domains will be critical for refining MTX-based strategies to improve the overall management of patients with IBD.

## Conflict of interest

Tommaso L. Parigi has received speaker fees from AbbVie, Dr Falk, Fresenius Kabi, Ferring, Janssen, Takeda, and Tillots and travel grants from AbbVie, Alfasigma, Janssen, CADI Group, Pfizer, Takeda. All other authors declare no conflicts of interest.
